# The Role of Small Molecules Containing Fluorine Atoms in Medicine and Imaging Applications

**DOI:** 10.3390/ph17030281

**Published:** 2024-02-22

**Authors:** Emily Henary, Stefanie Casa, Tyler L. Dost, Joseph C. Sloop, Maged Henary

**Affiliations:** 1 School of Science and Technology, Georgia Gwinnett College, 1000 University Center Lane, Lawrenceville, GA 30043, USA; ehenary@ggc.edu (E.H.); jsloop@ggc.edu (J.C.S.); 2 Department of Chemistry, Petit Science Center, Georgia State University, 100 Piedmont Avenue SE, Atlanta, GA 30303, USA; scasa1@gsu.edu (S.C.); tdost18@gmail.com (T.L.D.); 3 Center for Diagnostics and Therapeutics, Georgia State University, 100 Piedmont Avenue SE, Atlanta, GA 30303, USA

**Keywords:** fluorine, dye, fluorophore, chromophore, pharmaceutical, imaging

## Abstract

The fluorine atom possesses many intrinsic properties that can be beneficial when incorporated into small molecules. These properties include the atom’s size, electronegativity, and ability to block metabolic oxidation sites. Substituents that feature fluorine and fluorine-containing groups are currently prevalent in drugs that lower cholesterol, relieve asthma, and treat anxiety disorders, as well as improve the chemical properties of various medications and imaging agents. The dye scaffolds (fluorescein/rhodamine, coumarin, BODIPY, carbocyanine, and squaraine dyes) reported will address the incorporation of the fluorine atom in the scaffold and the contribution it provides to its application as an imaging agent. It is also important to recognize radiolabeled fluorine atoms used for PET imaging in the early detection of diseases. This review will discuss the many benefits of incorporating fluorine atoms into small molecules and give examples of fluorinated molecules used in the pharmaceutical industry and imaging techniques.

## 1. Introduction

The value of fluorine-containing small molecules with pharmaceutical efficacy and the underlying importance of fluorine incorporation into these molecules is undeniable [1,2]. Every year, new fluoro-pharmaceuticals achieve FDA approval and are introduced into the market; this is an ongoing trend that has been observed over the past 20 years [3,4]. In 2021, nine fluorine-containing drugs were approved for use by the FDA, showing an increase in relevancy for the incorporation of fluorine in medicinal research [5]. The design, synthesis, and testing of medically viable fluorine-containing compounds have burgeoned over the past two decades, leading to numerous publications and patents.

**Figure 1:**
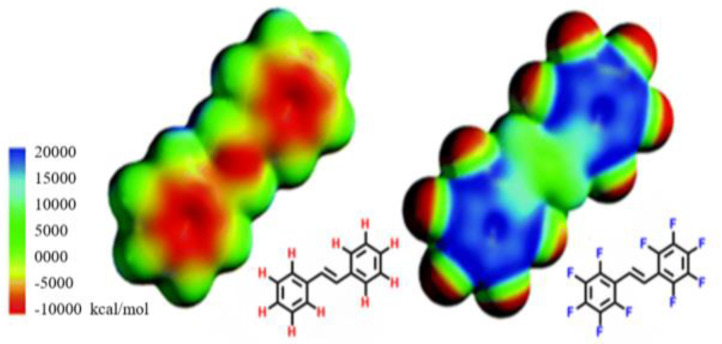
The relative sizes of stilbene and decafluorobenzene visualized through Spartan calculations. Figure used with permission [10]. Copyright 2005 Royal Society of Chemistry.

Utilization of the fluorine atom and fluorine-containing functional groups in pharmaceuticals is very attractive to the medical field for a variety of reasons [2]. First, the fluorine atom is the second smallest “functional group” with a van der Waals radius of 1.47 Å [1,6]. Because fluorine’s size falls between that of a hydrogen atom (1.20 Å) and an oxygen atom (1.47 Å), it is often used as a bioisostere for these atoms [6,7,8]. It has been suggested that the trifluoromethyl group is comparable to an isopropyl group, even though its van der Waals volume is much smaller (-CF_3_: 39.8 Å^3^ vs. -CH(CH_3_)_2_: 56.2 Å^3^) [1]. The fact that the -CF_3_ substituent has rotational symmetry around the carbon–carbon bond, whereas the isopropyl group lacks rotational symmetry, helps account for this apparent discrepancy [9]. As shown in Figure 1, modeling studies reveal the relative similarity in sizes between stilbene and perfluorinated stilbene while highlighting the dramatic impact fluorine has on electrostatic potentials in the stilbene molecule [10]. Because the fluorine atom is the smallest halogen, an additional advantage is found in its ability (as well as fluorinated methyl groups) to fit into smaller pockets of space in comparison to the other halogens and their corresponding groups.

Fluorine’s high electronegativity, 4.0 on the Pauling scale, offers it other advantages over similarly sized atoms and functional groups when incorporated into small molecules. The C-F bond is considered the strongest bond in organic chemistry due to the electronegativity difference between the two atoms [11,12]. In addition, the highly electronegative nature of fluorine often alters the dipole moment of the overall molecule. Another molecular property affected by the electronegativity of the fluorine atom is the pKa [13]. The acidity of fluorinated molecules increases due to inductive effects brought about by fluorine’s electronegativity [7,14].

**Figure 2:**
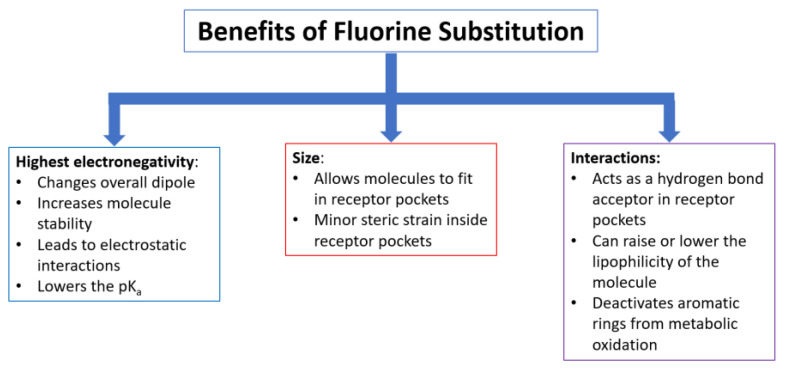
Effects of fluorine incorporation on drugs and imaging probes.

Additionally, benefits arising from the incorporation of one or more fluorine atoms into a compound often include alterations in drug biodistribution and drug–receptor binding, as well as enhancement of potency [15]. A significant advantage found upon fluorine substitution in drugs and imaging agents is the ability to modulate lipophilicity by adding or taking away fluorine atoms from the molecule in medical and biomedical imaging applications [16]. The tuning of lipophilicity helps the compounds to be absorbed and transported in vivo faster and more easily. This stems from the greater lipophilicity of the C-F bond relative to the C-H bond [17]. Because the fluorine atom is the smallest halogen, it (as well as fluorinated methyl groups) fits into receptor pockets and it contributes to blocking sites from metabolic oxidation more than that of other halogens [18]. The electronegativity and lipophilic properties of fluorine atoms incorporated into medicinal compounds have strong impacts on how the body will react to the molecules in ways such as the clearance rate, biodistribution route, and toxicity of the molecule. Figure 2 summarizes the key effects that fluorine substitution has on small molecules with potential pharmaceutical value.

Before introducing examples of fluorinated small molecules and their applications in medicine and imaging techniques, we will first discuss several key reactions by which fluorine and fluorinated functional groups are incorporated into compounds.

## 2. Common Reactions Incorporating Fluorine in Small Molecules

**Figure 3:**
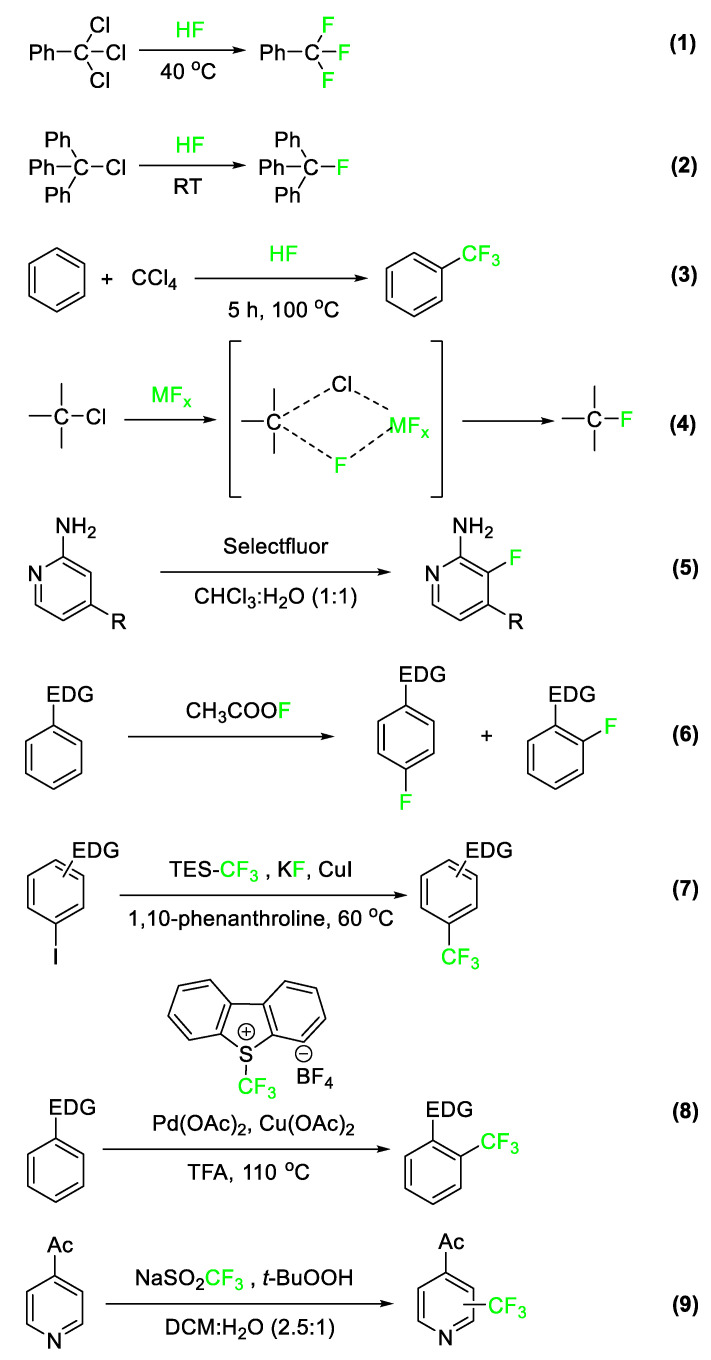
Examples of fluorination and trifluoromethylation reactions involve benzylic, aromatic, alkyl, and heteroaromatic substrates [9,10,11,17,20,22,23,24,26,27].

The introduction of fluorine and other fluorinated functional groups into the final drug architecture is most often accomplished via reactions with fluorinated precursors. Therefore, it is important to understand the reactions that are used to prepare these fluorinated precursor molecules. In Figure 3, a selection of processes used to prepare fluorobenzylic, fluoroalkyl, fluoroaromatic, and fluoroheteroaromatic systems is presented. For a more detailed discussion on current state-of-the-art organofluorine synthetic strategies, a review by Gouverneur et al. published in 2021 is illustrative [19]. The first reaction is performed at a large scale in industrial settings and involves hydrogen fluoride (HF) (Equation (1)). In this reaction, the fluoride anion serves as a nucleophilic substitute for other halogens occupying allylic and benzylic positions. [20]. Another reaction commonly used to incorporate fluorine is the Friedel–Crafts (FC) alkylation. Hydrogen fluoride, in this case, acts as both the FC catalyst and the fluorinating agent itself, resulting in the preparation of trifluoromethylated aromatic systems (Equation (2)). The carbon tetrachloride reagent in Figure 3, Equation (3), replaces a hydrogen atom on the benzene ring, followed by the substitution of fluorine for the three chlorine atoms.

Equation (4) is an example of the Swarts reaction. In this process, a metal fluoride species acts as a Lewis acid catalyst in removing a halide substituent and replacing it with a fluorine atom via the formation of a four-membered cyclic intermediate [21]. Equation (5) shows a green, highly regioselective fluorination of activated 2-aminopyridines using Selectfluor^®^ [22]. Equation (6) shows electrophilic fluorination using acetyl hypofluorite (CH_3_COOF) as the fluorinating reactant [23].

Equations (7) and (8) are examples of electrophilic trifluoromethylations of activated aromatic and heteroaromatic substrates developed within the last two decades [20,24]. Equation (7) employs trifluoromethyltriethylsilane (TES-CF_3_) under Cu(I) catalysis to prepare trifluoromethylated aromatic and heteroaromatic molecules from the corresponding iodide starting material. The mechanism by which this reaction takes place is not fully understood but may involve a CF_3_-Cu complex. Equation (8) depicts aromatic trifluoromethylation by S-(trifluoromethyl)dibenzothiophenium tetrafluoroborate via a transition metal-catalyzed reaction. This reaction generates a product with the trifluoromethyl group introduced to the ortho position of benzene [25]. Finally, Equation (9) is an example of a highly regioselective heteroaromatic trifluoromethylation of a pyridine derivative [26]. In the next section, we discuss a number of the more important fluorinated pharmaceuticals. In addition to elaborating on the role that fluorine plays in altering molecular properties, we will also note how and when fluorine and functionalities containing fluorine are introduced into the drug’s molecular architecture.

## 3. Characteristics of Pharmaceuticals Featuring Fluorine and Examples

Fluorinated therapeutics represent compounds available as drugs that use fluorine atoms to change the molecule’s overall lipophilicity, drug potency, and other factors. For many potentially viable pharmaceutical compounds, the clearance rate and clearance mechanism are critical considerations when designing and synthesizing molecules. Due to the extraordinary properties exhibited by the fluorine atom, drug designers continue to incorporate this atom into lead compounds. In the next section, the benefits of the properties of fluorine and fluorine-containing functional groups will be discussed.

### 3.1. Metabolic Oxidation

A primary reason to incorporate the fluorine atom, especially on aromatic and heterocyclic ring systems, is to combat the effect of metabolic oxidation which occurs when drugs are taken into the body and the process eliminates foreign molecules from the body as quickly as possible. The addition of a fluorine atom or fluorine-containing group often stops this metabolic pathway as the modified fluorobenzene does not fit into the active site of the monooxygenase. An additional benefit to adding fluorine to the para position on the benzene ring is that the inductive electron-withdrawing effect of the fluorine atom deactivates other positions on the ring against this particular metabolic pathway [9]. Slowing the oxidation process that the P450 protein performs allows for longer drug retention and thus increases effectiveness. Examples in the literature show compounds that have very rapid bodily clearances, but when fluorine is incorporated into the para position on a phenyl ring, the rate at which the compound is cleared slows by as much as 108-fold [28]. And, while the main function of the P450 protein is to initiate the metabolic process, oxygenizing a pharmaceutically active compound can turn it toxic [29]. Fluorine and chlorine can block this process from happening, but due to its minimal size, electronic withdrawing nature, and steric perturbation in active sites, fluorine is preferred over chlorine. Many therapeutics highlighted in this review article share the theme of a *p*-fluorophenyl group. The protection afforded by the *para*-substituted halogen reduces the potential toxicity when oxygenized and can increase drug effectiveness due to interactions with activation sites in proteins [9,30]. The strategy of *p*-fluorination has been thoroughly investigated and has been frequently used in drug design [29].

### 3.2. Electronic Considerations

While deactivating a drug against metabolic oxidation is possibly the most important consequence of incorporating fluorine into drugs, researchers can utilize fluorine atoms to alter compounds to achieve other strategic effects. Electronic considerations such as electronegativity, pKa modification, and lipophilicity tailoring are also motives that chemists take advantage of when designing fluorine-containing pharmaceutical compounds [4,15]. Electronegativity leads to several underlying effects on a molecule, including molecule and bond stability, dipole magnitude and direction, and electrostatic interactions with receptor sites. Here, several drugs are described that do not contain fluorine atoms for their metabolic effects but rather for their other properties.

### 3.3. Size Considerations

As mentioned previously, the size of fluorine-containing functional groups is very unique. Structurally, they take up more room than hydrogen but less than a hydroxyl group. The size of the functional groups can help direct a compound into its target pocket. Once the molecule is inside the pocket, the electronic characteristics of the fluorine then assist in adding to the potency and effectiveness of the drug.

### 3.4. Examples of Pharmaceuticals Containing Fluorine

**Figure 4:**
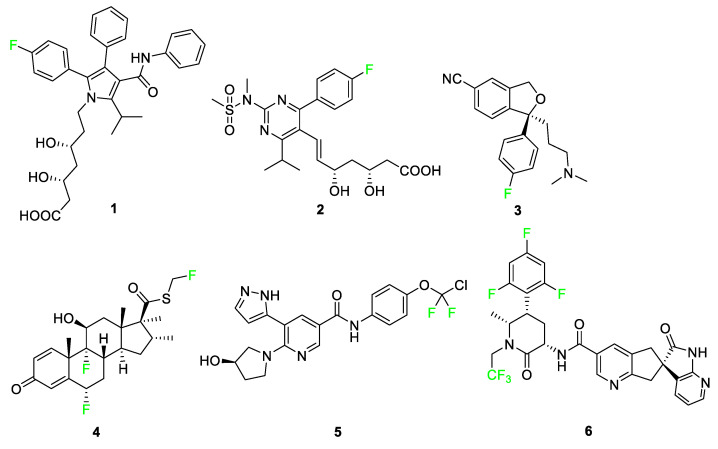
Six successful pharmaceutical drugs featuring fluorine [4,5,36,37].

Numerous pharmaceuticals feature fluorine, including six examples seen in Figure 4: atorvastatin (Lipitor) **1**, rosuvastatin (Crestor) **2**, escitalopram (Lexapro) **3**, fluticasone (Flonase^®^) **4**, asciminib (Scemblix^TM^-AB001) **5**, and atogepant (Qulipta^®^) **6**. These drugs treat high cholesterol, depression, anxiety disorders, allergic rhinitis, chronic myelogenous leukemia (CML), and migraines, respectively [3,31]. These six drugs are used by patients in every demographic, and their functions affect millions of people worldwide; however, these compounds represent only a fraction of pharmaceuticals that contain at least one fluorine atom. In this section, pharmaceutical compounds will be described and categorized in detail, paying special attention to the features of the compounds containing fluorine atoms highlighted in Figure 4.

Atorvastatin **1** and rosuvastatin **2** are statin drugs used in the treatment of patients exhibiting high cholesterol [32]. Rosuvastatin **2**, was developed from a desire to replace large, complex functional groups with simpler, achiral alternatives. The addition of the pyrimidine ring compared to other synthetic statins, such as atorvastatin **1**, improves activity for inhibiting the targeted reductase. The *p*-fluorophenyl group on this molecule not only enhances its biological activity but also deactivates the ring against P450 monooxygenase.

Escitalopram **3** is a selective serotonin reuptake inhibitor used to treat major depression and anxiety disorders [33]. The drug targets the serotonin transporter and facilitates the reuptake of serotonin into the neurons for rapid antidepressant activity [34]. A binding study of 5-HT transporter (SERT) was conducted to determine affinity for the two potential binding sites and substituent selectivity [35]. The study determined that derivatives of escitalopram containing aromatic fluorine and the presence of the cyano group were significant for a twofold decrease in dissociation rate and had a high contribution to allosteric potency.

Fluticasone **4** is used for relieving nasal symptoms such as sneezing, itching, and runny nose provoked by allergies; recent studies use this compound in combination with other medications to improve its effects against allergic rhinitis [38,39,40]. Also, fluticasone is combined with salmeterol and prescribed under the medication named Advair for the treatment of asthma as well as other obstructive airway diseases [41,42]. Fluticasone **4** containing the electron-withdrawing fluorine atom demonstrated greater topical activity compared to other analogs and increased glucocorticoid and mineralocorticoid effects [43].

Asciminib **5** is the active ingredient used in the treatment of CML containing a chiral compound with a difluorinated moiety [44]. CML is a disease associated with the overproduction of white blood cells in the bone marrow that arises from the BCR-ABL protein mutation leading to abnormal signaling pathways resulting in the growth and generation of leukemic cells [36,45]. These types of tyrosine kinase inhibitors work by binding to the ATP-binding site of BCR-ABL, thus transforming the disease into a controllable state during treatment [36,46]. The fluorine atoms in asciminib **5** interact with the carbonyl carbon of leucine-359 of the active site pocket, making the position of the fluorine atoms advantageous for improving the bioactivity of the overall compound [5].

Atogepant **6** is an orally administered calcitonin gene-related peptide (CGRP) receptor antagonist that aids in the treatment of migraines [37,47]. Substitution of the 1,3,5-trifluorobenzene moiety in place of a nonfluorinated benzene ring produces a four-fold increase in the affinity of atogepant **6** compared to the nonfluorinated benzene ring derivative [48,49,50].

**Figure 5:**
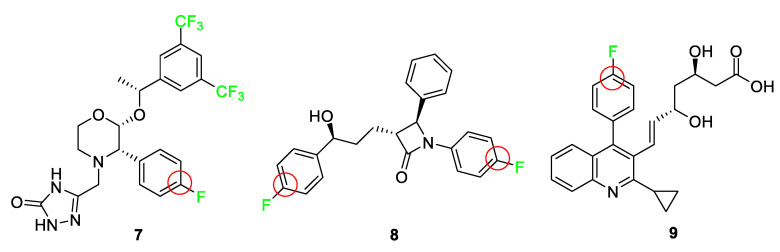
The drugs aprepitant **7**, ezetimibe **8**, and pitavastatin **9** with a fluorine atom and trifluoromethyl groups strategically block potential metabolic oxidation sites, highlighted by the red circles [51,53,54,55].

Approved by the FDA in 2003, aprepitant (Emend) **7** combats chemotherapy-induced nausea and vomiting and serves as an example where the addition of a fluorine atom blocks oxidative metabolism and lowers the oxidation potential elsewhere on the ring (Figure 5) [10,51]. Ongoing clinical trials show promising results for the use of this drug in cancer treatment [52]. The 3,5-bis-(trifluoromethyl)phenyl group is a common feature with other NK1 receptor antagonists and improves penetration of the drug into the central nervous system [51]. The fluorine atom blocks the 4-position of the benzene ring from oxidation as well as deactivates the remainder of the ring positions.

Ezetimibe (Zetia^®^) **8** [53] is a drug that combats high cholesterol by targeting Niemann–Pick C1-like 1 protein (NPC1L1), thus lowering low-density lipoprotein (LDL); it features two *p*-fluoro substituents to block other sites on the ring from metabolic oxidation and improves metabolic stability [12,56]. Figure 5 shows the structure of ezetimibe 8 and highlights the blocking of sites because of fluorine atoms. The compound utilizes the fluorine atoms as a defense against aromatic hydroxylation as well as yielding a derivative that exhibits improved pharmacokinetic properties, which increases the activity of the drug significantly. Another property of the fluorine atom substitution that ezetimibe **8** takes advantage of is the increase in polarity of the overall molecule. Molecules with higher polarity are more susceptible to glucuronidation, a process that generally inactivates drugs. However, in the case of ezetimibe **8**, glucuronidation improves the activity of the drug by recirculating the drug to the activation site and increasing the residence time [21]. Therefore, through the utilization of fluorine, researchers are able to increase lipophilicity and polarity, as well as block the oxidation sites for ezetimibe **8**, which in turn exhibited a 50-fold increase in activity over the parent compound.

Synthetic statins are a class of drugs that must contain 4-fluorophenyl groups as structural requirements for biological activity. Examples include atorvastatin **1**, rosuvastatin **2**, and pitavastatin (Livalo) **9**, as shown in Figure 4 and Figure 5. These drugs combat cholesterol problems by inhibiting hydroxymethylglutaryl-coenzyme A (HMG-CoA) reductase. All of these compounds feature a *p*-fluorophenyl moiety, as studies have shown that this substituent greatly surpasses the biological activity of all other functional groups tested [54,55]. Pitavastatin **9**, a recent statin-type drug to enter the market, is completely synthetic. This HMG-CoA reductase inhibitor and low-density lipoprotein cholesterol (LDL-C) receptor inducer improves its pharmacokinetics, efficiency, and bioavailability by incorporating a *p*-fluoro group [57]. The 2-cyclopropyl-4-(4-fluorophenyl)quinoline pharmacophore differentiates it from other statins, and the highly functionalized heterocycle provides superior resistance to metabolism and prolongs its duration of action [58]. A modified version of pitavastatin **9** has been used for positron emission tomography (PET) imaging in vivo by incorporating a fluorine-18 atom [59].

**Figure 6:**
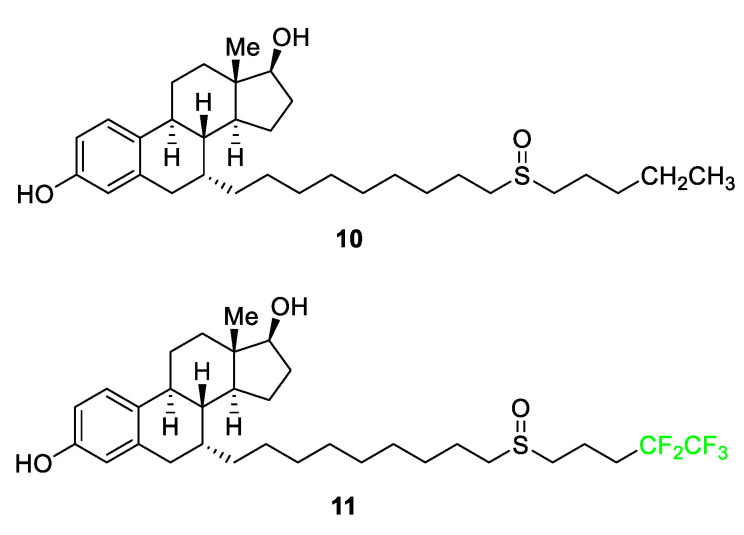
The parent molecule **10** and the FDA-approved drug derivative, fulvestrant **11** [60].

As seen in Figure 6, fulvestrant (Faslodex) **11** utilizes the replacement of an *n*-ethyl functional group with a pentafluoroethyl moiety [60]. ICI 164,384 **10**, the original precursor, was developed for the treatment of breast cancer, specifically to combat the negative side effects of the receptor modulator Tamoxifen which increased the risk of the metastasis of associated tumors. The parent molecule did not display high levels of potency during in vivo testing. The introduction of the -CF_2_CF_3_ group in step 1 of the 12-step synthesis of compound **11**, resulted in a five-fold increase in intrinsic potency relative to the parent molecule **10** [60]. The addition of the terminal pentafluoroethyl group as opposed to the ethyl end-chain increases the strength of the bonds and makes the compound more stable as well as increases hydrogen bonding with the receptor, thus increasing metabolic stability during estrogen receptor (ER) binding [60].

**Figure 7:**
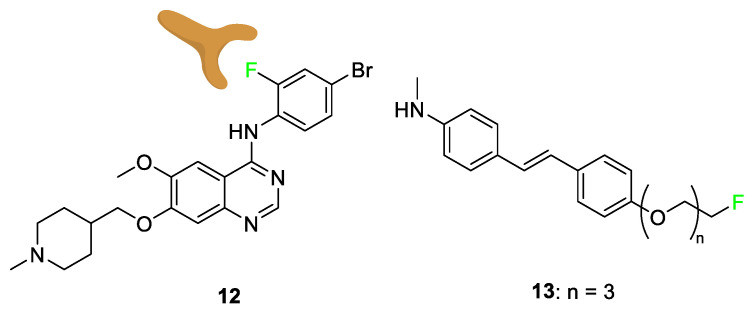
Vandetanib **12** and AV-45 derivative **13** use the fluorine atom to tailor the lipophilicity of the overall compound or certain parts of the compound [66,67].

Multiple drugs use the added lipophilicity afforded by the substitution of a hydrogen atom with a fluorine atom; lipophilicity alteration can lead to greater drug uptake through cell membranes [61,62,63,64]. While fluorination of an aromatic or *π*-system increases lipophilicity, the addition of fluorine and trifluoromethyl groups on an *n*-alkyl chain does not have the same effect; some tailoring and chemical designs of aliphatic fluorination can decrease lipophilicity [65]. Vandetanib (Caprelsa) **12** [66], a drug that acts as an antagonist of the vascular endothelial growth factor receptor (VEGFR), is used as an oral kinase inhibitor for thyroid tumors. This is an example of a compound modified with fluorine to achieve the “Goldilocks” level of lipophilicity (Figure 7). Many derivatives of vandetanib **12** were tested to tailor the lipophilicity of the compound to increase its potency. Structure–activity relationship tests show that bromine at the C-4’ position was preferred, and that fluorine was optimal at the C-2’ position. The bromofluorophenyl group was analyzed as a residue matching these two cases, and it was shown that the fluorine atom leads the bromofluorophenyl group deep into the protein’s hydrophobic pocket, ultimately increasing the potency of the drug [66].

Compound **13**, a specific AV-45 derivative with three additional polyethylene glycol (PEG) groups added to the compound, was tested alongside other chain lengths to determine how varying the hydrophilicity altered efficacy [67]. These compounds were being studied for amyloid beta (Aβ) plaque affinity in relation to combatting Alzheimer’s disease. Compound **13** displayed good Aβ binding coupled with high blood–brain barrier penetration. A range of fluoroethylene glycol (FPEG) lengths was studied to find the greatest uptake depending on the logP (lipophilicity). The result shows only a minimal change in uptake when modifying the PEG length below n = 5. However, when the FPEG group was replaced with a hydroxyl group, a significant increase in lipophilicity and, more importantly, a decrease in potency was observed [67].

**Figure 8:**
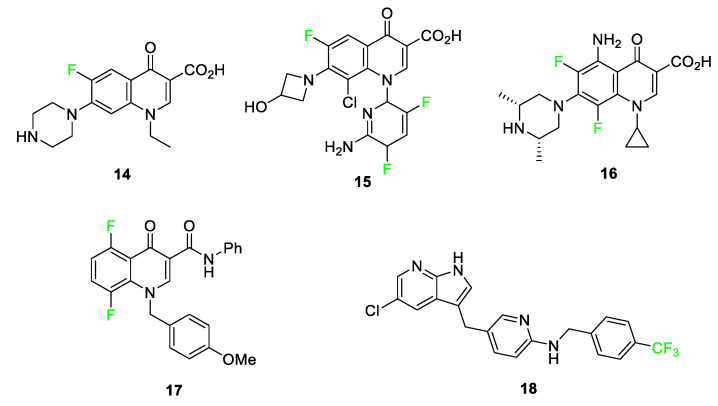
Norfloxacin (Noroxin) **14**, delafloxacin (BAXDELA^®^) **15**, sparfloxacin (Zagam) **16**, 5,8-difluoro-1-(4-methoxybenzyl)-4-oxo-N-phenyl-1,4-dihydroquinoline-3-carboxamide **17**, and pexidartinib (TURALIO^®^) **18** [69,70,71].

Fluorine-containing quinolones, pyrimidoquinolines, and pyridyl-substituted indoles exhibit anti-cancer properties [68]. The fluorine atom and trifluoromethyl substituents play major roles in the anti-cancer, antimicrobial, and antituberculosis effectiveness of these compounds [69,70]. Adding fluorine to this class of small molecules increases the hydrophobicity of the compounds, which in turn, aids in the penetration into hydrophobic protein pockets. Examples of common amino acids that attract the hydrophobic fluorine group include leucine and phenylalanine [68]. More specific examples of these compounds **14**–**18** are shown in Figure 8.

**Figure 9:**
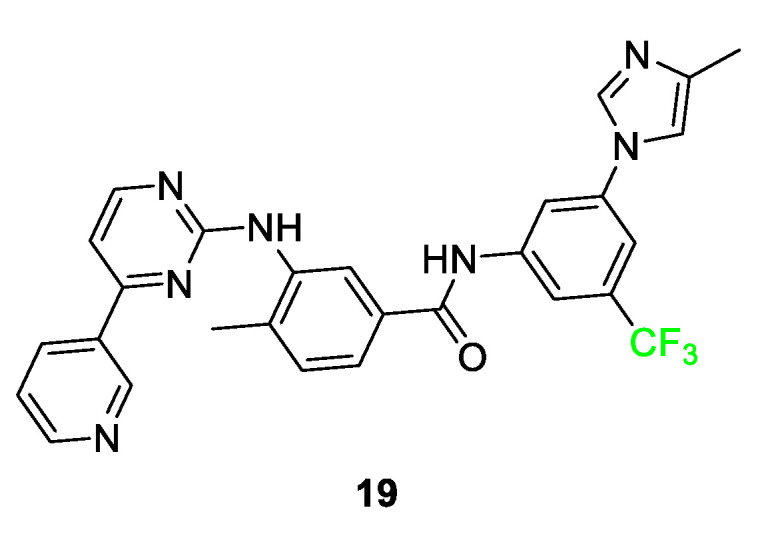
Nilotinib **19** is a compound that inhibits a tyrosine kinase inhibitor in patients with chronic myelogenous leukemia [72].

In Figure 9, nilotinib (TASIGNA^®^) **19**, a derivative of Imatinib, is shown. The addition of the trifluoromethyl group resulted in the compound exhibiting 30 times the potency when compared to the nonfluorinated parent compound. Once inside the pocket of the Bcr-Abl tyrosine kinase inhibitor, the trifluoromethyl group interacts with the histidine and isoleucine side chain residues. By comparison, when an analog featuring a methyl substituent was tested in place of the trifluoromethyl, it showed a five-fold decrease in activity relative to compound **19** [55,72].

**Figure 10:**
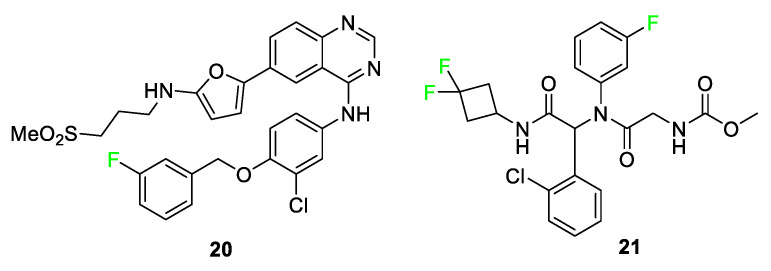
Lapatinib **20** and ivosidenib **21** utilize fluorine as an essential size requirement for potency [73,74].

Other compounds that utilize the size of fluorine to advantage are lapatinib (TYKERB^®^) **20** and ivosidenib (TIBSOVO^®^) **21** (Figure 10) [73,74]. Lapatinib **20** is a human epidermal growth factor inhibitor and dual tyrosine kinase inhibitor for fighting breast cancer and other solid tumors. Groups larger than the featured *m*-fluorophenyl, e.g., the m-chlorophenyl group, displayed diminished drug activity for compound **20** analogs. Analogs that substituted -OH or -Br in place of the m-fluorophenyl group showed substantial decreases in inhibition. These findings led researchers to the conclusion that fluorine is essential to fit into the binding pocket as well as to the interactions that retain the drug in the pocket; X-ray crystallographic results support this conclusion [73]. Ivosidenib **21** is a drug developed to treat relapsed or refractory acute myeloid leukemia in adults with an isocitrate dehydrogenase 1 (IDH1) mutation. Fluorine substitution on the phenyl ring brought about desirable metabolic stability in observations from compound AGI-14100 containing two aromatic fluorine atoms; however, in the same study, compound **21** was determined to have more desirable properties upon changing the design to incorporate a nitrogen atom in the ring containing fluorine atom [74].

Thus far, this review article has highlighted therapeutic agents and pharmaceutical compounds that feature fluorine atoms or fluorine-containing functional groups on small molecules. This special atom has become extremely important in drug development in recent decades and will continue to be an essential building block when designing and synthesizing drugs [4,75,76]. The characteristics of fluorine and the benefits of incorporation on pharmaceutical molecules, whether they be size considerations, electronic properties, or blocking of metabolic sites susceptible to the P450 monooxygenase, are undeniable in medicinal chemistry.

**Figure 11:**
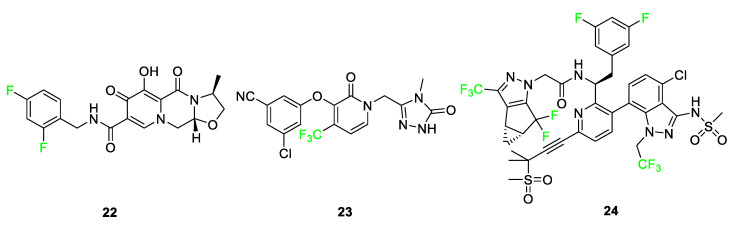
Fluorine-containing FDA-approved drugs for use in HIV treatment: cabotegravir **22**, doravirine **23**, and lenacapavir **24** [14,79].

In the literature, fluorinated compounds including cabotegravir (Vocabria) **22**, doravirine (PIFELTRO™) **23**, and lenacapavir (SUNLENCA^®^) **24** are recent FDA-approved drugs that have been used in the development of HIV treatments (Figure 11) [14]. Cabotegravir **22** has been used in conjunction with rilpivirine as the cocktail under the name of Cabenuva [5,36,44]. The introduction of a fluorine atom to the benzene ring of cabotegravir compared to its analog improved its potency as an agent. In 2021, a study was conducted where it was used as an injectable in combination with rilpivirine to give it a long-lasting effect as treatment. Doravirine **23**, like rilpivirine, is a non-nucleoside reverse transcriptase inhibitor (NNRTI) used in HIV treatment after it was FDA-approved in 2018 [47]. Its structure is a trifluorinated compound compared to an existing chlorine-containing analog; the modification to the structure shows improvements in half-life and plasma stability in studies conducted. Lenacapavir **24**, FDA-approved in 2022, is used as a capsid protein inhibitor with pico-molar range potency and it showed significant viral suppression after some weeks when administered to patients. The introduction of fluorine to the structure anchors the conformation, thus improving the drug’s potency [47].

**Figure 12:**
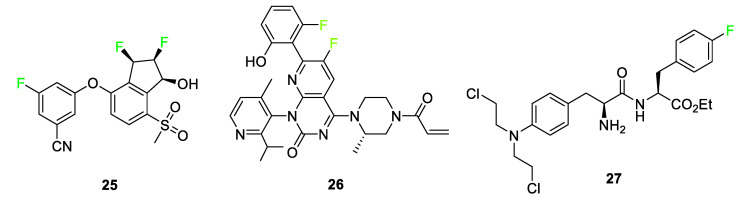
Anti-cancer medications containing fluorine atoms: belzutifan **25**, sotorasib **26**, and melphalan flufenamide **27** [5,36].

Anti-cancer medications are also highlighted as a sector where fluorinated compounds have been introduced (Figure 12). Belzutifan (WELIREG™) **25** is a hypoxia-inducible factor-2α inhibitor used for the treatment of Hippel–Lindau disease which is associated with the appearance of renal cell carcinoma, hemangioblastomas, and/or pancreatic neuroendocrine tumors [36]. Sotorasib **26** (LUMAKRAS^®^), an elective and irreversible covalent inhibitor B, is used to treat non-small cell lung cancer [36]. Sotorasib **26** interacts with a cysteine-12 residue of KRAS mutation, KRas G12C, to disrupt the signaling pathway. This KRAS mutation brings about the following tumors: lung adenocarcinoma, pancreatic ductal carcinoma, and colorectal carcinoma. The fluorine-containing version of the compound overcame the bioavailability issue observed for derivatives of the compound containing different halogens [5]. Melphalan flufenamide (Pepaxto^®^) **27**, a drug approved in 2021 for use in the treatment of myeloma, is 10 times more pharmacologically active than melphalan [5,77]. The presence of the *p*-fluoro substituent on melphalan flufenamide **27** enhances metabolic stability over nonfluorinated melphalan analogs [78].

**Figure 13:**
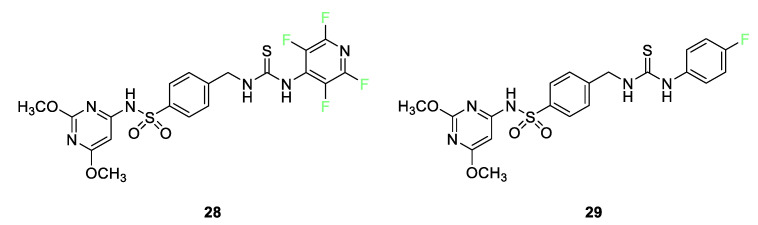
Tetrafluoropyridyl sulfonamide **28** and parafluorophenyl sulfonamide **29** [80].

The bioactivities of a venerable class of compounds, sulfonamides, have seen a resurgence in research interest over the past decade [80]. Numerous examples of fluorinated sulfonamides show enhanced biological efficacy over their nonfluorinated counterparts. The enhancements in effectiveness can arise from resistance to oxidation, electronic alterations, or size brought about by fluorine substitution. Tetrafluoropyridyl sulfonamide **28** (Figure 13) was found to be active against both Gram-positive (*B. subtillis* and *S. pneumoniae*) and Gram-negative (*P. aeruginosa* and *E. coli*) bacteria strains as well as effective against fungi (*A. fumigatus*). This sulfonamide was also active in a cytotoxic assay against the MCF-7 breast cancer and HepG2 hepatic cancer cell lines. Activity exceeded that of both cisplatin and 5-fluorouracil. Molecular docking shows that sulfonamide exhibits good interactions with amino acid residues on the mitogen-activated kinase active site. The parafluorophenyl sulfonamide **29** was found to be active in a cytotoxic assay against the HepG2 hepatic cancer cell lines with activity on par with cisplatin. Molecular docking shows that sulfonamide **29** exhibits good interactions with amino acid residues on the mitogen-activated kinase active site.

**Figure 14:**
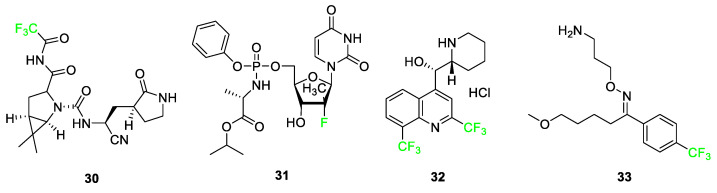
COVID-related fluorine-containing drugs: nirmatrelvir **30**, sofosbuvir **31**, mefloquine HCl **32**, and fluvoxamine **33** [36].

Some fluorinated drugs have been found to be relevant in the treatment of patients with mild to moderate symptoms of severe acute respiratory syndrome coronavirus 2, SARS-CoV-2. Nirmatrelvir (PAXLOVID™) **30** is a protease inhibitor containing a -CF_3_ group that is used to disrupt virus replication (Figure 14) [36]. In COVID-19 patients, it has been used alongside ritonavir to maintain the concentration of nirmatrelvir **30** during treatment. This combination of drugs was the first orally administered treatment approved by the FDA for the treatment of COVID-19. Other drugs such as ensitrelvir (Xocova^®^) and favipiravir (Avigan) are two promising antiviral compounds currently undergoing studies for potential use in the treatment of SARS-CoV-2.

Fluorinated drugs used to treat other diseases have been repurposed for potential uses in the treatment of SARS-CoV-2 such as sofosbuvir (SOVALDI^®^) **31**, mefloquine HCl (Lariam^®^) **32**, and fluvoxamine (Luvox^®^) **33**, In 2013, sofosbuvir was used as a pro-antiviral drug for the treatment of the Hepatitis C virus, and in 2020, it was being studied for inhibition of RNA-dependent RNA polymerase in SARS-CoV-2 replication. Mefloquine HCl **32** was originally used as an antimalarial drug; studies conducted in 2021 adopted this drug for usage in the treatment of COVID-19 as the fluorinated version of the molecule increased its antiviral activity compared to derivative hydroxychloroquine (Figure 14). Fluvoxamine **33**, originally used for obsessive-compulsive disorder (OCD) as a selective serotonin reuptake inhibitor, is being studied for its effect in reducing the need for hospitalization in COVID-19 patients [36].

**Figure 15:**
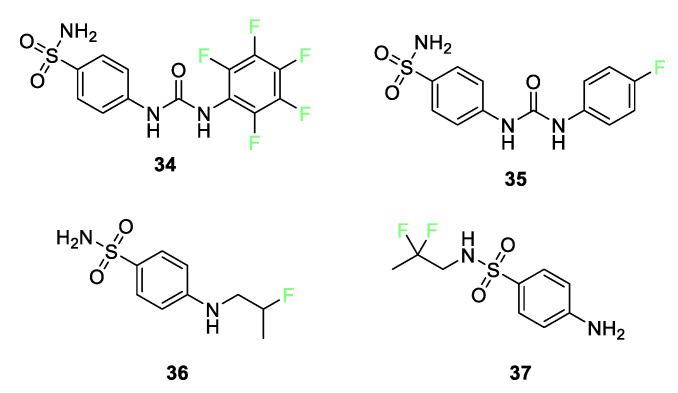
Fluorine-containing sulfonamides is used for inhibition of carbonic anhydrase.

In a recent review, Berrino et al. recounted the current state of research into the enhancement of carbonic anhydrase (CA) inhibition by fluorination of sulfonamides [81]. Several research teams have investigated [82,83,84,85] how aromatic fluorine substitution (compounds **34** and **35**) as well as fluoroalkyl substitution (compounds **36** and **37**) can improve CA II and CA IX inhibition (Figure 15).

The presence of fluorine in the para position of the aromatic rings in compounds **34** and **35** makes oxidation unlikely. As discussed earlier, in vitro inhibition studies show that compounds **34** and **35** inhibit the tumor-associated CA IX [82,83,84,85]. Compound **35** is in Phase 1b/II clinical trials as a CA IX inhibitor (with gemcitabine) for hypoxic solid tumor treatment. The fluorine(s) give the aryl group pharmacokinetic and pharmacodynamic properties not enjoyed by the nonfluorinated analogs. Additionally, X-ray studies of compound **35** have shown that the larger hydrophobic pocket of CA IX can better accommodate the tail of compound **35**, leading to efficient hydrophobic interactions.

Substitution of fluorine on alkyl chains attached to either the aniline nitrogen or the sulfonylamino group can also enhance CA inhibition. Substitution of a 2-fluoropropyl moiety on the aniline nitrogen in compound **36** strongly enhances the inhibition potency relative to its nonfluorinated N-allylic precursor against CA II by a factor of 40 and against CA IX by a factor of 30. When the fluorinated chain is attached to the sulfonyl nitrogen, as in compound **37**, the difluoro group enhances the inhibition of CA II and CA IX by a factor of 40.

## 4. Fluorine Incorporated into Dye Imaging Agents

In addition to pharmaceuticals, another important medicinal chemistry application in which fluorine has found wide use is in imaging agents. Where the previous compounds were synthesized to fight and inhibit disease, the next section will focus on compounds that image and detect diseases. Imaging, both preoperative and in vivo, is another sector of medicine that has become a popular technique in recent years to assist in the fight against many diseases. First, we will present several classes of dyes that can be used for in vivo imaging and then consider several examples of fluorine-containing compounds that have been used in preoperative imaging. Fluorinated probes can illuminate several regions of the electromagnetic spectrum. Before presenting specific examples of fluorinated imaging agents, we will describe the benefits and shortcomings of a few different classes of biological imaging probes, including coumarin, fluorescein/rhodamine, boron–dipyrromethine (BODIPY), and cyanine dyes.

The electromagnetic spectrum spans from high-energy gamma rays to low-energy radio waves. The visible light spectrum lies between these extremes and is usually defined as having wavelengths of 500–600 nm. Dyes in this region are generally [86] used for surface imaging due to the higher probability of native tissue absorption in this range [87] when compared to near-infrared (NIR) dyes that absorb light at longer wavelengths. Because of this, very few examples of probes for biological imaging exist in this range. Dyes that fluoresce in this range can be used for purposes other than biological/medical applications, including solar cell construction.

### 4.1. Coumarin Dyes

**Figure 16:**
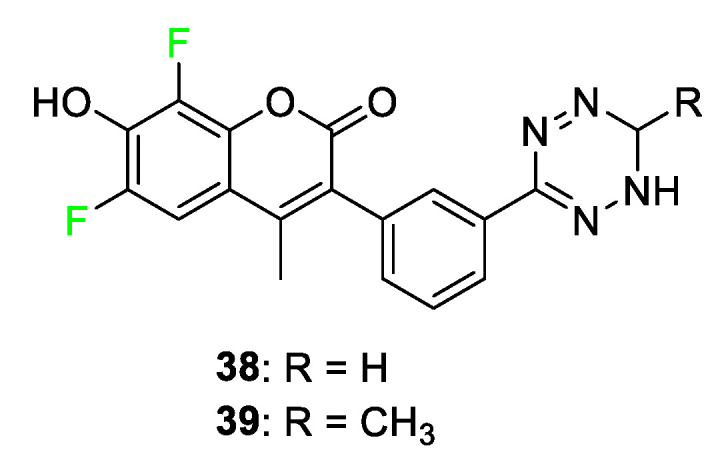
Two fluorinated coumarin dyes, **38** and **39**, fluoresce near 450 nm [89].

The coumarin class of dye is a blue light-absorbing dye. Coumarin fluorophores are utilized for the diagnosis and imaging of diseases such as cancer. Coumarin molecules are small and biocompatible, and have a relatively high light quantum yield compared to other fluorescent dye classes [88]. Two examples of fluorine-incorporated visible light coumarin probes were discovered by Weissleder [71] and coworkers. The dyes, shown in Figure 16, contain two fluorine atoms on the coumarin derivatives **38** and **39**. The coumarin dyes that contain fluorine substituents, in comparison to those without, show very comparable extinction coefficients, 19,000 to 16,000, while having a higher quantum yield, 49% to 41%, respectively [89]. The dyes effectively label the biological targets as well as feature optical flexibility and fast reactivity with the targets [89].

### 4.2. Fluorescein/Rhodamine Dyes

Fluorescein and rhodamine dyes consist of two important groups: the xanthene moiety, which acts as the fluorophore, and the benzene moiety, which provides the photoinduced electron transfer (PeT). PeT is a known mechanism in which the chromophore’s fluorescence is quenched with the electron transfer from the benzene donor to the xanthene acceptor [90,91]. The unsubstituted fluorescein dye **40** has a carboxyl group at position 2 of the benzene moiety, and until recently, it was believed that this acceptor group was essential to the molecule; this is based on results replacing the COO- group with hydrogen, reducing the quantum yield value by over 60% [92]. These xanthene-based dyes are seen as highly tunable and, therefore, are used as biological markers for DNA and proteins. Xanthene dyes do have some flaws, including being very pH-dependent; the forms in which the compounds can exist range from neutral to dianionic. A second shortcoming of the xanthene class of dyes stems from the wavelengths that these dyes absorb and fluoresce under 600 nm because there is high interference from tissue autofluorescence below 600 nm [93]. However, due to the high quantum yield and fluorescence tunability of these dyes, many of them still find use as biological markers, although their use as DNA stains is not performed in vivo [90,91].

**Figure 17:**
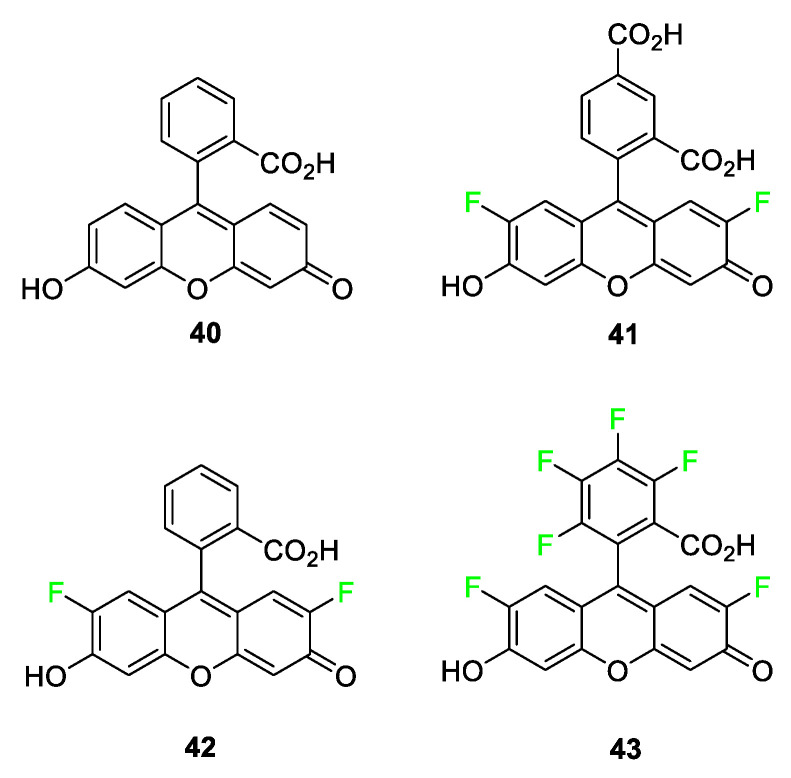
Fluorescein **40** and fluorine-containing derivatives **40**–**43** show decreased photobleaching and comparable quantum yield [94].

Upon synthesizing a host of fluorinated fluorescein derivatives (Figure 17), it was found that these compounds exhibited some very interesting characteristics. While exhibiting slightly less molar absorptivity in comparison to the parent compound **40**, the quantum yields of selected fluorinated xanthenes increased to nearly 100%. Another exciting fluorine-induced feature combats one of the drawbacks of this class of compounds as a whole: photobleaching. The bleaching value dropped from 17 (fluorescence percentage loss after 33 min) to as low as 4. It is hypothesized that this phenomenon likely stems from the triplet state lifetime of the molecule. The fluorine atoms, at the 2’ and 7’ position, in compounds **41**–**43**, shorten the triplet lifetime so that the likelihood of reaction with its quencher is decreased [94]. Although these compounds have not yet been tested in vivo, compounds **41** and **43** show great promise for use in bioconjugation in the future. When comparing these compounds’ absorbance wavelengths, compounds **41** and **42** are the same while a notable 18 nm redshift is observed for compound **43**. Fluorophore **43** has a longer wavelength emission compared to fluorescein **40**, and the report mentions its chemical properties (pKa, photostability, and high quantum yield), making it a useful compound for future investigation.

**Figure 18:**
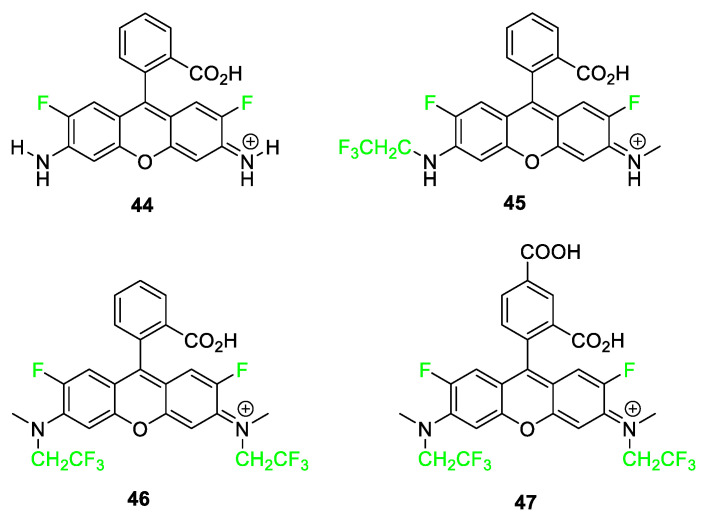
Rhodamine derivatives featuring fluorine and trifluoroethyl groups, compounds **44**–**47** [95].

Rhodamine dyes **44**–**47** have similar benefits and use in molecular imaging as the xanthene dye moiety as well as similar shortcomings (Figure 18). The core structure of rhodamine differs only in the two amine groups in place of the carbonyl and hydroxyl groups on the xanthene. This structural feature creates the limitation of pH dependency because the compounds can exist in neutral, zwitterionic, cationic, or dicationic forms. As in the case of the fluorinated fluorescein dyes, researchers likewise discovered an increased quantum yield with the fluorinated rhodamine dyes, along with prolonged photostability and decreased photobleaching of the dyes. Compounds **44** and **46** have similar absorbance and emission wavelengths; however, they differ in that compound **46** with two CH_2_CF_3_ groups attached to nitrogen has a higher reported quantum yield than compound **44**. Hell and coworkers synthesized several rhodamine analogs featuring at least two fluorine atoms and discovered that this class of dyes shows great promise for stimulated emission depletion (STED) nanoscopy, a technique used for elucidating protein structures [95].

### 4.3. Boron–Dipyrromethene Dyes

The near-infrared region of light on the electromagnetic spectrum lies between 700 and 1000 nm. This region of light presents a unique range that allows for various uses in biomedical imaging. Because of the specific range in which the dyes absorb and fluoresce, NIR fluorophores avoid several potential problems concerning body imaging that were present with the coumarin, xanthene, and rhodamine dyes. Body tissue inherently fluoresces light in the 450–500 nm range, a phenomenon known as autofluorescence. Thus, imaging agents that fluoresce in this region exhibit a high background signal. The use of an NIR filter essentially eliminates tissue autofluorescence altogether [96] This makes higher-wavelength-fluorescing molecules superior in terms of signal-to-background noise ratios and molecular brightness. The upper end of the NIR range (near 1000 nm) is of limited usefulness due to water overtones that begin in this region. NIR light also has the ability to penetrate tissue for several centimeters [97]. This is due to the lower tissue absorbance and reduced scattering. Because of these factors, NIR fluorophores present themselves as the best potential in vivo imaging agents.

**Figure 19:**
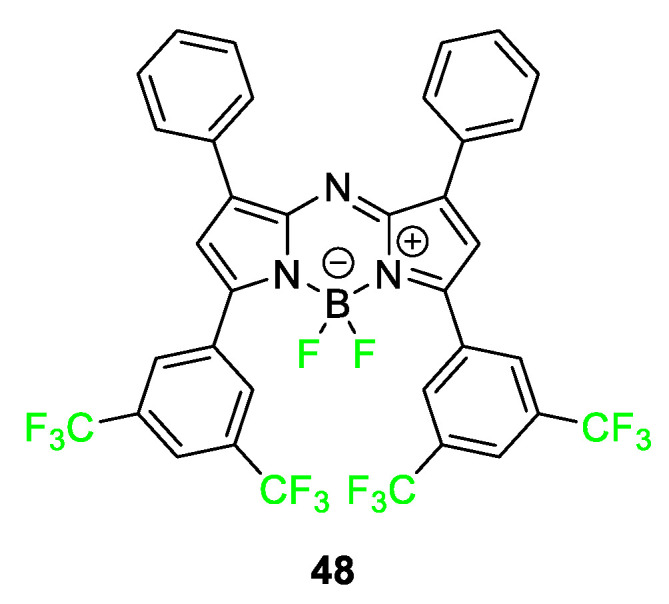
The structure of aza−BODIPY dye **48** [98].

Many different classes of NIR fluorophores contain fluorine atoms. One of the most common types is BODIPY dye. The core structure of the BODIPY dye contains two fluorine atoms in the structure with a host of possible alteration sites. In recent studies, aza-BODIPY dyes have become increasingly popular in the literature due to their extended conjugation giving them more favorable optical properties for imaging [16]. Figure 19 features aza-BODIPY **48** highlighted in 2022 demonstrating dual imaging capabilities and potential for use in photodynamic therapy [98].

**Figure 20:**
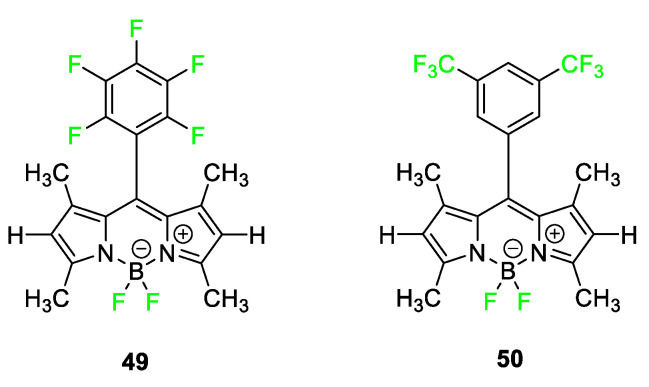
Examples of BODIPY dyes **49** and **50** feature perfluorophenyl or 3,5−ditrifluoromethylphenyl groups on top of the two fluorine atoms already on the molecule [99].

Although all BODIPY dyes feature fluorine attached to their core boron, very few BODIPY dyes further incorporate fluorine into the molecular architecture. Figure 20 outlines two examples, **49** and **50,** which contain a perfluorophenyl ring and *m*-ditrifluoromethylbenzene ring, respectively, but the dyes were not tested for in vivo imaging [99]. The perfluorophenyl ring dye **49** has been used in studies to observe its reaction with thiols and amines, making them potential fluorophores for XPS/fluorescence labeling [100].

### 4.4. Carbocyanine Dyes

One class of NIR fluorophores that has yet to feature many lead compounds containing the fluorine atom for biological activity is the carbocyanine dye family. This class of dyes is often utilized in biomedical imaging. Several benefits arise from using carbocyanine dyes as an in vivo imaging agent. First, carbocyanine dyes are non-toxic to humans. In fact, Indocyanine green, a dye currently FDA-approved and used for medical imaging, remains one of the least toxic agents ever to be administered to humans [96]. Additionally, the core structure of the carbocyanine dye can easily be modified to match the wavelength needed: each double bond added between the indolium end units contributes to a wavelength increase of around 100 nm. While adding more carbon to the molecule does increase hydrophobicity, these dyes also have the advantage of having many different sites of modulation. This hydrophobicity increase caused by a longer carbon chain can be offset by adding a sulfonate or carboxylate group or another hydrophilic functional group to any of these sites.

**Figure 21:**
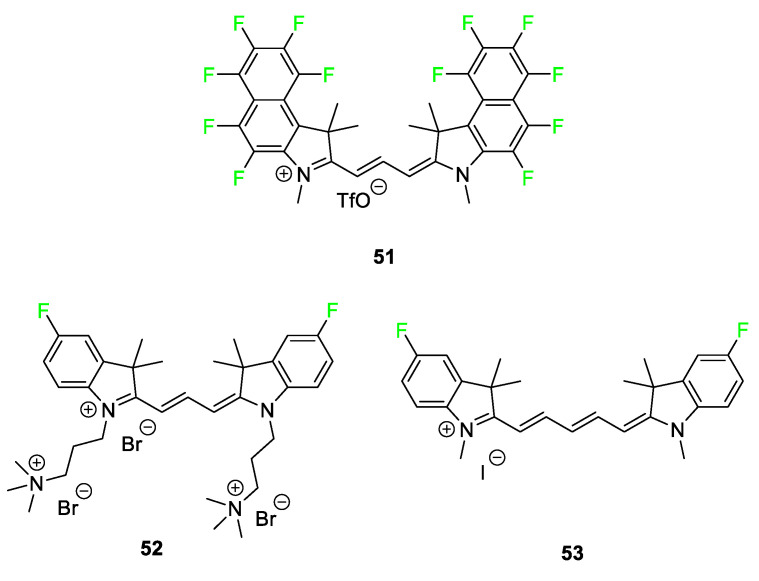
Representative fluorinated carbocyanine dyes **51**−**53** [17,101,102].

Very few examples of carbocyanine dyes featuring the fluorine atom exist in the literature. Three examples of fluorocarbocyanine dyes are shown in Figure 21. Dye **51** is a fluorous amine-sensitive cyanine dye that was modified to observe fluorescence changes caused by the introduction of fluorine atoms to a known trimethine dye [101]. The results demonstrate that ratiometric and colorimetric property changes are observed upon exposure to amines. Dye **52** [102] selectively binds to G-quadruplex DNA; compound **53** [17] targets the thyroid and parathyroid glands. The fluorinated analog of the G-quadruplex DNA binding dye **52** shows a large increase in the thermal stability of the telomeric quadruplex. For the endocrine-targeting carbocyanine dyes, the uptake of the fluorinated molecule **53** was more than double that of any other substituent studied in the article, including other halogens and electron-donating groups. The fluorinated compounds exhibited a slower bodily clearance rate and were still observed in vivo after 4 h. The promising nature of these compounds for intraoperative imaging has led researchers to begin exploring these dyes as dual-modal molecules.

### 4.5. Squaraine Dyes

A significant portion of previous literature examples focuses on the use of squaraines in dye-sensitized solar cells (DSSCs). [103,104,105]. Squaraine dyes are growing in interest to the scientific community as many research groups are now finding biological applications for this dye scaffold [106,107]. One of the most promising properties of squaraine dyes is the relatively high quantum yield associated with this dye family compared to others: squaraine dyes are reported to have quantum yields between 20 and 40% [108]. They are also expected to have excellent molar absorptivity and high photobleaching thresholds. However, one of the problems related to squaraine dyes is the molecular stability around the oxycyclobutenolate ring in the center of the linker. Nevertheless, new examples of symmetrical and asymmetrical squaraine dyes have been synthesized and have demonstrated promising stability [105,109,110].

**Figure 22:**
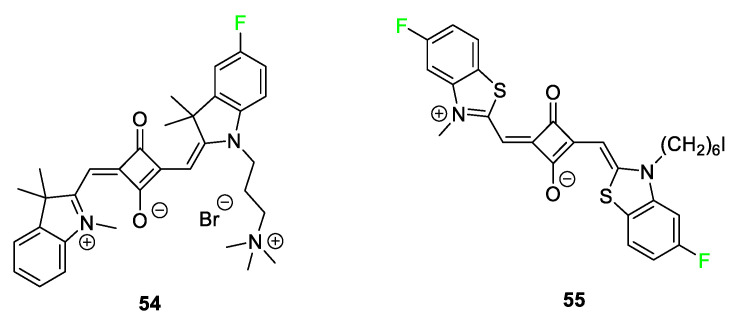
Representative fluorinated squaraine dyes used for biological applications [111,112].

Figure 22 highlights a squaraine dye **54** [111] used for imaging ovarian cancer. This imaging agent stood out in cytotoxicity and NIR bioimaging studies when compared to analogs containing hydrogen, chlorine, or bromine atoms. Squaraine dye **55** [112] is used for labeling oligonucleotides. When compared to a nonfluorinated corresponding squaraine dye or dicyanosquaraine dye, the fluorine-containing dye **55** demonstrated improved photophysical properties and chemical stability.

## 5. Fluorine Incorporated into Molecules for Early Disease Detection Imaging Agents

Due to the increased success rate of both surgery and chemotherapy during the early stages of tumor development, a need for enhanced detection methods has emerged. Visualization of diseased tissues gives doctors and surgeons insight into the answers to important questions such as: where is the diseased tissue, how big is the diseased mass, and what is the best course of action to combat the issue? Depending on the stage at which some diseases are detected, visualization of diseased tissues leads to curative measures. [97,113]. Currently, many imaging methods are used in the healthcare industry, including positron emission tomography (PET). These imaging methods aid healthcare professionals in assessing a patient’s specific situation and defining a course of action. Imaging agents can be used that will bind to specific tissues as well as give off energy in the form of positrons as emission radiation or radiative return, which is how the instrument obtains the visualization. While agents such as tagged proteins, nanoparticles, and metal delivery systems have been explored for use in these types of imaging modalities, small organic molecules are becoming popular for PET imaging.

### Fluorine in PET

PET scans are presently among the most sensitive molecular imaging technique modalities [97]. The ^18^F-fluorine isotope has great utility in PET imaging due to its relatively long half-life (110 min.) in comparison to other positron-emitting atoms [114]. Additionally, fluorine-18 decays into oxygen-18, a non-toxic, non-radioactive nucleus that, when conjugated onto a sugar, is excreted through the kidneys and liver [53]. These advantages have driven the development of safe fluorinated contrast agents in use today [114].

**Figure 23:**
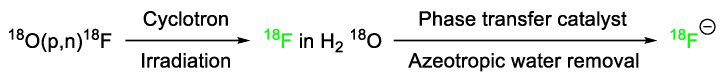
Production of ^18^F−fluoride.

Production of fluorine-18 requires a cyclotron, which irradiates ^18^O with protons. If the irradiation target is liquid H_2_^18^O, an aqueous solution of ^18^F-fluoride results. The ^18^F-fluoride is then treated with a suitable salt such as the tetrabutylammonium cation or phase transfer catalyst such as Kryptofix_2.2.2_. The water is then removed by azeotropic distillation [115]. See Figure 23.

**Figure 24:**
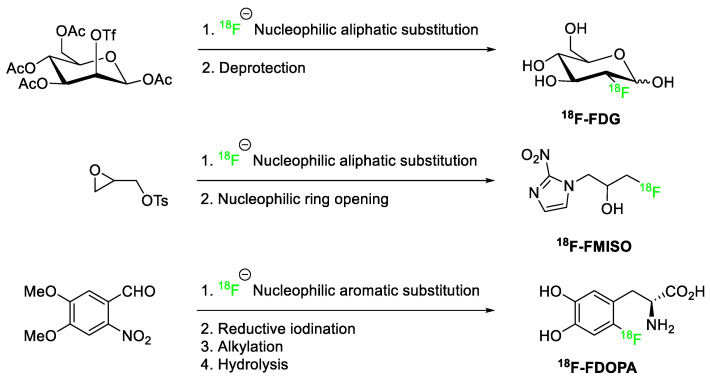
Selected ^18^F−aliphatic and ^18^F−aromatic nucleophilic substitutions [117,118,119,120].

The ^18^F-fluoride may then be incorporated into molecules by nucleophilic aliphatic substitution (S_N_2) reactions or nucleophilic aromatic substitutions (S_N_Ar). One advantage to this incorporation strategy is the high specific activity of fluoride-18 (~100 GBq/μmol) [116,117]. Figure 24 depicts several examples of radiotracers produced by nucleophilic substitutions using ^18^F-fluoride as the nucleophile.

The efficiency of S_N_2 nucleophilic substitutions depends on several factors, including substrate topology, leaving group ability and solvent choice. Ideally, substrates which allow for the nucleophile to approach the carbon bearing the leaving group (primary benzylic > primary aliphatic > secondary aliphatic) with minimal steric interference are preferred. Additionally, the selection of a satisfactory leaving group is paramount; the leaving group should facilitate the nucleophilic substitution [115]. In the examples depicted in Figure 24, both the triflate (OTf) and tosylate (OTs) leaving groups used in the preparation of ^18^F-FDG and ^18^F-FMISO, respectively, are exceptionally well suited as leaving groups [115]. Polar aprotic solvents are typically used for nucleophilic substitutions. In the figure above, acetonitrile (CH_3_CN) [118] served as the solvent to prepare ^18^F-FDG while dimethyl sulfoxide (DMSO) was the solvent used to prepare ^18^F-FMISO [119].

For nucleophilic aromatic substitutions (S_N_Ar) to be successful, the aromatic ring system must contain electron-withdrawing groups positioned to enable mesomeric stabilization of the negative charge upon initial addition of the nucleophile. In the reaction depicted in Figure 24, the ^18^F-fluoride attacks the carbon bearing the -NO_2_ group (a typical leaving group for S_N_Ar reactions). The aldehyde group ortho to the nucleophilic site of attack stabilizes the negative charge by resonance. The polar aprotic solvent dimethyl formamide (DMF) was the medium in which ^18^F-FDOPA was prepared [120].

While ^18^F-fluoride nucleophilic substitution reactions remain the preferred method for [^18^F]-labeled radiotracer production [116], electrophilic methods have been explored to enable C-^18^F bond formation in a variety of [^18^F]-labeled compounds that are used diagnostically [50,114,121,122,123,124,125,126,127,128,129,130,131]. Recent, thorough reviews of methodologies to form C-^18^F bonds by Chen et al. [115], Zhiyi and coworkers [132], and Lui et al. [133] provide excellent accounts of the state of the art in the production of [^18^F]-labeled compounds.

Since [^18^F]-FDG was approved for PET by the US Food and Drug Administration in 1999, more than thirty ^18^F-tagged compounds have been developed and found to be efficacious in PET applications for a variety of medical maladies [133]. The extensive and diverse uses of ^18^F-labeled compounds in PET have been well documented over the last twenty years, with more than 30,000 PET-related research articles and reviews published in the last decade alone, as a review by Crișan et al. notes [134]. This field of study remains vibrant, and there is no doubt that compounds labeled with ^18^F-fluorine will find additional utility in the PET area of medicine.

## 6. Summary and Outlook

The utilization of fluorine atoms in chemistry has increased in the past 30 years, and its viability and importance in drug development and bioimaging have become readily apparent to scientists [16]. Fluorine’s many unique characteristics and properties lend themselves to the use of this atom in combatting the many different problems medicinal chemists may face. Throughout recent years, we have witnessed the impact of fluorine-containing compounds on different aspects of medicine including compounds being used as antivirals in HIV and COVID-19 patients as well as being used for bacterial studies and anti-cancer studies [14,36].

The inherent size, electronegativity, and blocking of metabolic sites for drugs, along with the shortening of the triplet state for imaging chromophores, can be solved singularly by the addition of one or more fluorine atoms on a molecule. Similarly, in medicine, the addition of fluorine atoms to contrast agents is becoming increasingly prevalent in biomolecular imaging and preventative medicine. Other forms of imaging incorporate fluorine atoms as a radiolabel for increased use of fluorescent probes in multimodal imaging. A unique opportunity presents itself for researchers when considering the addition of fluorine on small molecules or imaging fluorophores: multipurpose molecules. Not only are the fluorinated chromophores useful intraoperatively and for drug tracking in the body, but radiolabeled compounds also have utility as ^18^F-radiolabels for PET and SPECT purposes. Through the research performed on small molecules containing fluorine atoms and functional groups containing fluorine, much has been learned about drug metabolic pathways and medical advancements both through pharmaceutical and imaging techniques.

While many fluorinated compounds are strategically designed to alter the physical properties and metabolism of overall molecules, not all compounds necessarily benefit from fluorination. Some reports have addressed the possibility of fluorinated compounds degrading during metabolic processes and found that these active agents can generate reactive intermediates, thus creating potentially indirect in vivo toxicity [135]. While this toxicity is an undesirable effect, some studies have used these effects for generating fluorine-facilitated selective toxins expected to be useful as antibiotics and anti-cancer agents. Research trends on fluorinated compounds continue to increase. Considering research trends, we anticipate more compounds to be introduced into the pharmaceutical market; medicinal chemists must consider the medicinal effect of fluorine moieties but also look into the possible byproducts and overall effects when designing compounds. It is also important to consider the limitations of drug design based on insufficient fluorine-containing precursor availability. Several fluorinated compounds developed in earlier studies introduced single fluorine atoms into the chemical structures and explored their bioactivity and chemical property changes. Current studies are further expanding on original designs and exploring the incorporation of fluorine on multiple aromatic and aliphatic moieties, as well as the addition of chiral centers and their overall effects on molecules and their applications. It is imperative for researchers in the field to continue the development of these reagents to introduce new possibilities for incorporating fluorinated compounds into the field of medicine and imaging.

## Data Availability

Data sharing is not applicable.
